# Current hotspot and study trend of transcatheter aortic valve replacement, a bibliometric analysis from 2009 to 2023

**DOI:** 10.3389/fcvm.2025.1411561

**Published:** 2025-04-14

**Authors:** Ping Lai, Dekuan Zhang, Jin-hua Xue, Shuquan Xu, Kejun Tian, Hong-zhou Zhang, Bei Wang, Yi-ming Zhong, Yong-ling Liao

**Affiliations:** ^1^ Department of Cardiology, First Affiliated Hospital of Gannan Medical University, Gannan Medical University, Ganzhou, Jiangxi, China; ^2^ Key Laboratory of Prevention and Treatment of Cardiovascular and Cerebrovascular Diseases, Ministry of Education, Gannan Medical University, Ganzhou, China; ^3^ The First School of Clinical Medicine, Gannan Medical University, Ganzhou, Jiangxi, China; ^4^ School of Basic Medicine, Gannan Medical University, Ganzhou, China

**Keywords:** transcatheter aortic valve replacement, bibliometric analysis, research hotspot, CiteSpace, VOSviewer

## Abstract

**Introduction:**

Transcatheter aortic valve replacement (TAVR), alternatively termed transcatheter aortic valve implantation (TAVI), represents a seminal advancement in cardiovascular interventions by obviating the necessity for open-heart surgery traditionally associated with surgical aortic valve replacement (SAVR). This technique entails percutaneous delivery of a bioprosthetic valve. Despite the surfeit of literature on TAVR over the past fifteen years, a bibliometric analysis is conspicuously absent.

**Method:**

A query executed on the Web of Science Core Collection (WoSCC) on September 1, 2022, returned 8,359 articles and reviews pertinent to TAVR. Data interpretation leveraged Microsoft Excel, CiteSpace, and VOSviewer to illustrate trends and delineate focal points within the corpus of TAVR research.

**Result:**

The analysis incorporated 8,359 articles and reviews on TAVR from January 1, 2009, to August 1, 2023. Publication volume expanded from 35 in 2009 to a pinnacle in 2020, reflecting a near thirty folds increase, with citations escalating from 56 in 2009 to 27,354 in 2021. The United States prevailed in scholarly output (Np = 3,015), citation frequency (Nc = 70,991, excluding self-citations), and academic impact (H-index = 120). Columbia University was distinguished by the highest number of publications (Np = 380), citations (Nc = 41,051), and H-index (84). Within the author community, Rodes-Cabau J was preeminent, with 260 publications and an equivalent citation index and H-index. Keywords such as “balloon-expandable valve,” “coronary access,” “next-day discharge,” “conducti on disturbances,” and “coronary obstruction” have surfaced as the lexicon of burgeoning research themes.

**Conclusion:**

Investigation into TAVR has emerged as a major area of scholarly focus. The United States stands at the forefront of this research. Columbia University ranks as the preeminent institution in terms of publication output. Key research themes such as “balloon-expandable valve,” “coronary access,” and “coronary obstruction” are shaping up as current and prospective research hotspots, signaling potential areas for future study and innovation.

## Introduction

Transcatheter Aortic Valve Replacement (TAVR), also known as Transcatheter Aortic Valve Implantation (TAVI), has revolutionized cardiovascular medicine by offering a minimally invasive alternative to conventional surgical aortic valve replacement (SAVR) (1, 2). The evolution of TAVR has been marked by rapid advancement and sustained innovation (3). Initial efforts in the early 2000s culminated in the first human TAVR procedure in 2002, marking a pivotal moment in cardiac intervention (4). Initially developed for high-risk or inoperable patients, TAVR's indications have rapidly expanded to include intermediate- and low-risk individuals, fueled by advancements in procedural techniques and improved operator expertise (5, 6).

Early pivotal studies, such as the PARTNER and CoreValve trials, demonstrated TAVR's non-inferiority to SAVR for high-risk patients, leading to widespread clinical adoption (7, 8). Subsequently, the SURTAVI and NOTION trials further expanded TAVR's applicability to intermediate-risk populations, consolidating its role as a viable alternative to SAVR (9, 10). A landmark 2020 study confirmed TAVR's safety and effectiveness in patients with aortic stenosis, reinforcing its place in clinical practice (5). Modern valve designs improve hemodynamic performance and reduce paravalvular leakage (11, 12) and the miniaturization of delivery systems and advancements in imaging technologies, such as three-dimensional transesophageal echocardiography, have enhanced procedural precision, improving patient outcomes and reducing risks (13–15).

TAVR's versatility has expanded to include more complex cases, such as bicuspid aortic valve disease and pure aortic regurgitation, which were previously deemed unsuitable for the procedure (16–18). Additionally, the introduction of valve-in-valve procedures has provided a minimally invasive solution for patients with failing bioprosthetic valves, eliminating the need for repeat open-heart surgery (19). With over 8,000 studies published in the past fifteen years, TAVR's role in cardiovascular medicine continues to grow.

Bibliometric analysis is a methodological approach that has been reliably applied across various disciplines to delineate research trends and hotspots (20) —such as in the study of COVID-19, cardiac tissue engineering, and the role of gut microbiota in cardiovascular diseases (21–23), remains underutilized in the TAVR domain. This study aims to perform a comprehensive bibliometric analysis of TAVR-related literature, mapping its development, identifying current research hotspots, and projecting future directions. Through this analysis, we seek to capture the emergence of TAVR as a transformative innovation in cardiovascular medicine and highlight its enduring impact on the treatment of valvular heart disease.

## Material and Methods

Data for this investigation were sourced exclusively from the Web of Science Core Collection (WoSCC). The search parameters were as follows: TI (Title) = (“Transcatheter Aortic Valve Replacement” OR “transcatheter aortic valve implantation” OR “TAVR” OR “TAVI”). Inclusion criteria stipulated that only articles published in English from January 1, 2009, to August 31, 2023, were considered, yielding a total of 8,359 articles and reviews. The 2022 impact factor (IF) and Hirsch index (H-index) for these papers were acquired from the Web of Science portal, while citations per paper were referenced from the 2022 Journal Citation Reports (JCR).

Data collation and analysis were conducted using Microsoft Excel 2019. For the visualization and assessment of prolific countries, authors, co-cited authors, and highly cited publications, VOSviewer (version 1.6.18) was utilized. Further, CiteSpace (version 6.1.R3, 64-bit) was employed to identify and visualize the keywords demonstrating the strongest citation bursts and to map out the timeline view of keyword co-occurrence. The settings in CiteSpace were adjusted in accordance with our previous methodologies (21, 23). The methodology culminated in a flowchart that delineates the search strategy and article selection process, as depicted in Supplementary Figure S1.

## Results

### Overview of the research status in this field over the last fifteen years

**Figure 1:**
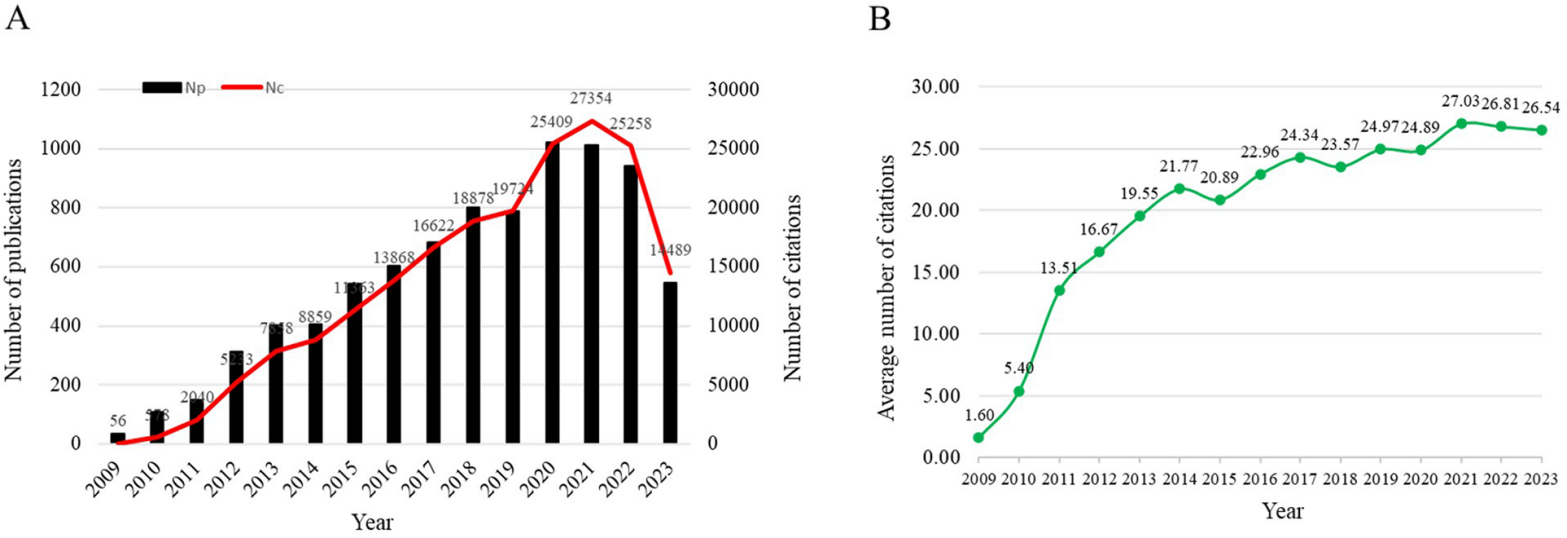
**(A)** The trend of publications and citations, and **(B)** the trend of average number of citations per year globally in the transcatheter aortic valve replacement from 2009 to 2023.

Over the last fifteen years, the field has experienced a significant publication boom, with 8,359 papers released. The increase in output is dramatic, soaring from 35 publications in 2009 to a peak in 2020, a near 30-fold rise. Citations have paralleled this growth, rocketing from 56 to a remarkable 27,354 in 2021—a more than 450-fold escalation (Figure 1A).

The trajectory of average annual citations is equally telling. From a humble average of 1.6 citations per paper in 2009, there was a marked increase to an average of 19.55 by 2013. Since 2014, this average has remained robustly above 20.0 citations per paper annually, reflecting a strong and continued interest in the domain. In the past three years, average citations have reliably hovered above 25.0, with 27.03, 26.81, and 26.54 for 2021, 2022, and 2023 respectively (Figure 1B).

### Countries or regions contributed all publications

**Figure 2:**
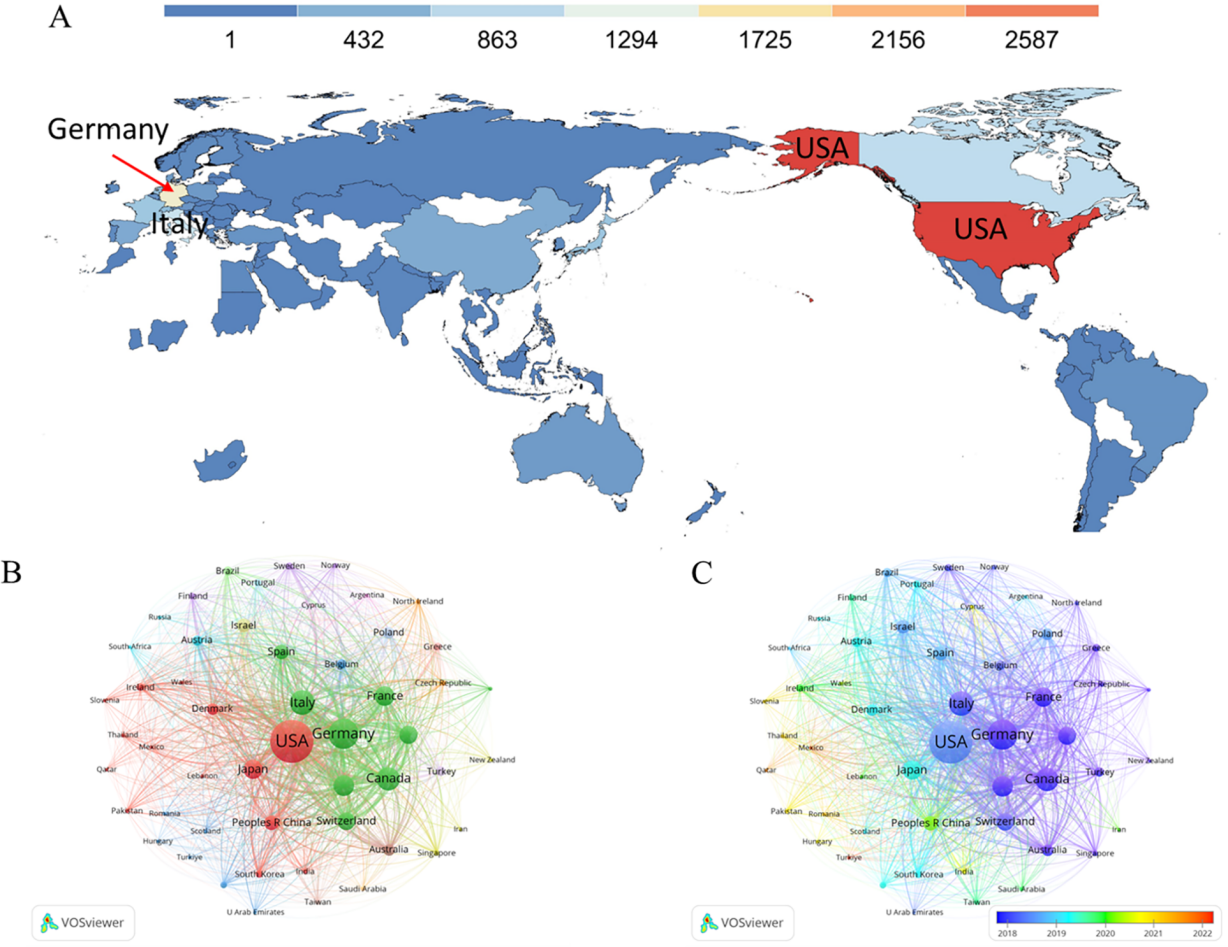
**(A)** Geographical distribution map of global publications related to transcatheter aortic valve replacement. Colors ranging from cold to warm represent an increasing number of publications. **(B,C)** Visual network of countries or regions with more than 10 papers. Each network node represents different country or region; the larger the node indicates the more publications. The thicker the line linking the nodes reflects the closer the cooperation between the countries or regions.

A total of 96 countries or regions contributed to all the papers displayed in Figure 2A. Remarkably, the top ten countries or regions collectively accounted for nearly half of these publications. The United States, taking the lead with 3,015 papers, an impressive 70,991 citations, and an H-index of 120, secured the top spot. Following closely were Germany (Np: 1,491, Nc: 36,779, H-index: 94) and Italy (Np: 989, Nc: 23,617, H-index: 77) (Table 1).

**Table 1:** Top Ten countries with highest number of publications in the field of TAVR.

Countries	Np	Nc	ACN	H-index
USA	3,015	70,991	23.55	120
Germany	1,491	36,779	24.67	94
Italy	989	23,617	23.88	77
Canada	897	59,751	66.61	117
England	686	32,217	46.96	73
France	639	23,701	37.09	82
Japan	609	5,177	8.50	37
Netherlands	546	21,862	40.04	71
Switzerland	509	16,089	31.61	66
Peoples R China	361	2,639	7.31	25

Np, number of publications; Nc, number of citations without self-citation; ACN, average cited number.

Additionally, it's noteworthy that 52 countries or regions had more than 10 papers to their credit (Figures 2B). Among these, Thailand, Qatar, Slovenia, Romania, and Turkiye emerged as new contributors, showcasing a keen focus on research related to this field (Figures 2C).

### Institutions participated to this field

**Figure 3:**
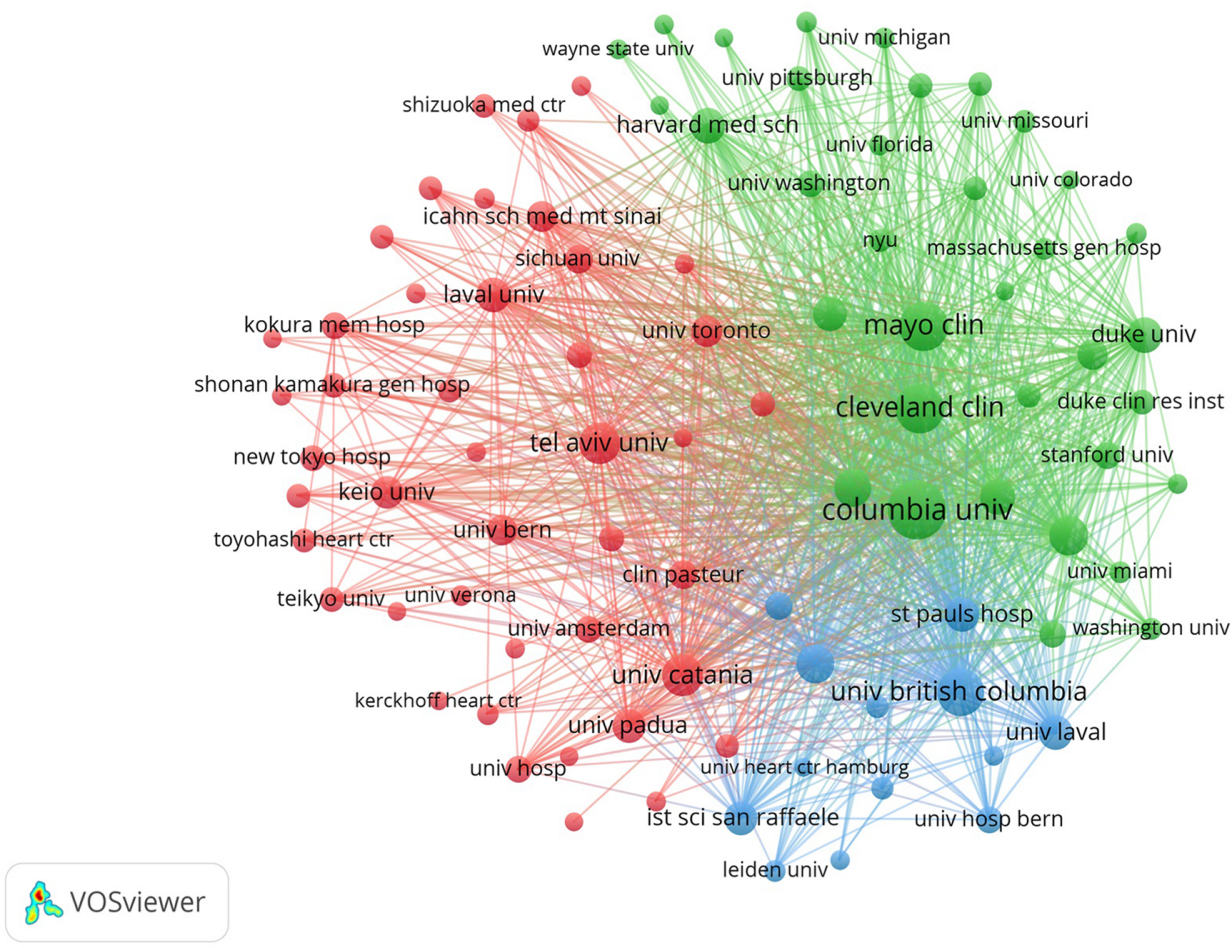
Visual network of 91 institutions owing more than 50 papers. Each network node represents a different institution; the larger the node means the more publications. The thicker the line linking the nodes represents the closer the cooperation between the institutions.

A total of 8,359 publications were collectively contributed by 6,167 institutions. Among these, 91 institutions demonstrated remarkable productivity by publishing more than 50 papers (Figure 3). Leading the pack, Columbia University, situated in the USA, emerged as the institution with the highest number of publications (Np) at 380, accompanied by an impressive 41,051 citations and an H-index of 84. The Cleveland Clinic Foundation (Np: 361) and Harvard University (Np: 361) secured the second and third positions, with 36,083 and 18,216 citations, respectively. Notably, among the top ten institutions with the most publications, St. Paul's Hospital (Np: 361) from Canada boasted the highest average citation count per paper, standing at an impressive 126.35. Of these top ten institutions, five are located in the USA, three in Canada, and the remaining two in France and Italy, respectively (Table 2).

**Table 2:** Top ten institutions with the most publications.

Institutions	Np	Nc	ACN	H-index	Country
Columbia University	380	41,051	108.03	84	USA
Cleveland Clinic Foundation	361	36,083	99.95	70	USA
Harvard University	361	18,216	50.46	55	USA
St Paul S Hospital	330	41,694	126.35	93	Canada
Udice French Research Universities	326	15,012	46.05	65	France
Laval University	308	23,281	75.59	78	Canada
Quebec Heart Lung Institute	292	19,036	65.19	78	Canada
Mayo Clinic	279	21,876	78.41	56	USA
Newyork Presbyterian Hospital	270	30,519	113.03	69	USA
Vita Salute San Raffaele University	261	12,407	47.54	59	Italy

Np, number of publications; Nc, number of citations without self-citation; ACN, average cited number.

### Authors contributed those papers

**Figure 4:**
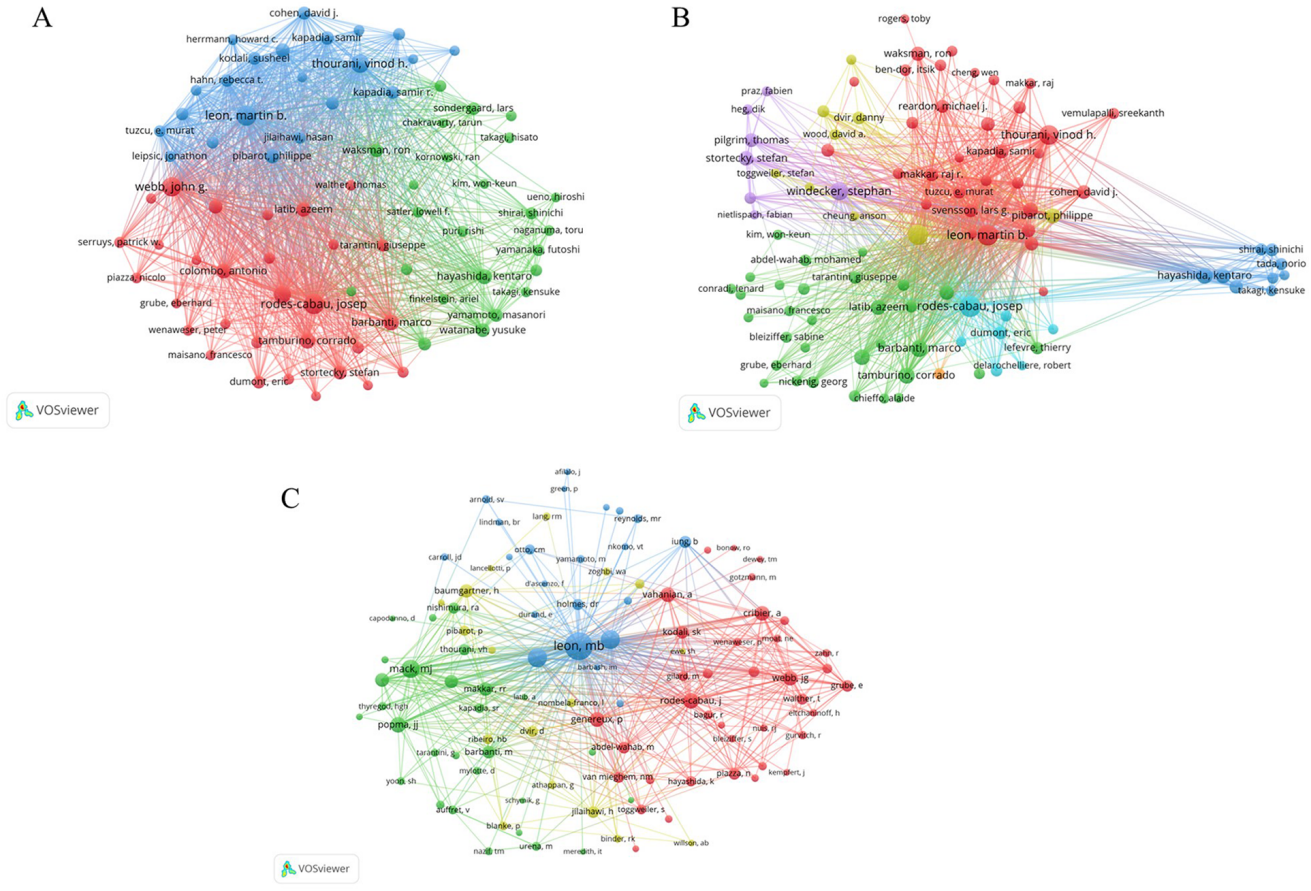
**(A)** Visual network map of 76 authors with more than 60 papers. **(B)** Visual network map of 97 authors producing over 50 papers accumulating more than 1,000 total citations. **(C)** Visual network map of 104 co-cited authors who have garnered more than 300 citations each. The node size stands for the number of citations each. Lines between nodes represent cooperation between authors.

In total, a grand total of 26,818 authors made contributions to all the papers within this field. In terms of the Np, Rodes-Cabau J (Np: 260, Nc: 19,381, H-index: 79), hailing from Spain, secured the top spot. Webb JG (Np: 238, Nc: 43,036, H-index:89) from Canada followed closely as the second most productive author, closely pursued by Leon MB (Np: 234, Nc: 41,455, H-index:81) from the USA (Table 3). Notably, among these prolific authors, 76 individuals have authored more than 60 papers (Figure 4A). Furthermore, 97 authors have distinguished themselves by producing over 50 papers, each accumulating more than 1,000 total citations (Figure 4B). Additionally, there are 104 co-cited authors who have garnered more than 300 citations each (Figure 4C).

**Table 3:** Top ten most productive authors in the field of TAVR.

Authors	Np	Nc	ACN	H-index	Country
Rodes-cabau J	260	19,381	74.54	79	Spain
Webb JG	238	43,036	180.82	89	Canada
Leon MB	234	41,455	177.16	81	USA
Barbanti M	212	9,853	46.48	56	Italy
Thourani VH	190	24,529	129.10	61	USA
Windecker S	185	15,259	82.48	55	Switzerland
Latib A	184	7,409	40.27	47	USA
Tamburino C	169	9,367	55.43	52	Italy
Colombo A	160	7,473	46.71	46	Italy
Sondergaard L	155	7,465	48.16	39	Denmark

Np, number of publications; Nc, number of citations without self-citation; ACN, average cited number.

### Journals contributed those publications

All publications included in the current study were published in 664 different journals. In terms of Np, “*Catheterization and Cardiovascular Interventions*” (Np: 616, Nc: 8,219, H-index: 43), followed by the “*American Journal of Cardiology*” (Np: 512, Nc: 9,900) and “*JACC Cardiovascular Interventions*” (Np: 369, Nc: 18,888) (Table 4).

**Table 4:** The top 10 journals with the most publication in the field of TAVR.

Journal	Np	Nc	ACN	H-index	IF	JCR
Catheterization And Cardiovascular Interventions	616	8,219	13.34	43	2.3	Q3
American Journal of Cardiology	512	9,900	19.34	51	2.8	Q3
JACC Cardiovascular Interventions	369	18,888	51.19	80	11.3	Q1
EuroIntervention	263	6,984	26.56	47	7.7	Q1
International Journal of Cardiology	227	4,033	17.77	33	3.5	Q2
Cardiovascular Revascularization Medicine	196	1,182	6.03	17	1.7	Q3
Journal of The American College of Cardiology	163	25,039	153.61	92	24	Q1
Journal of Invasive Cardiology	161	1,307	8.12	18	1.5	Q4
Frontiers In Cardiovascular Medicine	146	463	3.17	10	3.6	Q2
Circulation Cardiovascular Interventions	142	5,578	39.28	45	5.6	Q1

Np, number of publications; Nc, number of citations; ACN, average cited number; IF, impact factor; JCR, journal cited report.

**Figure 5:**
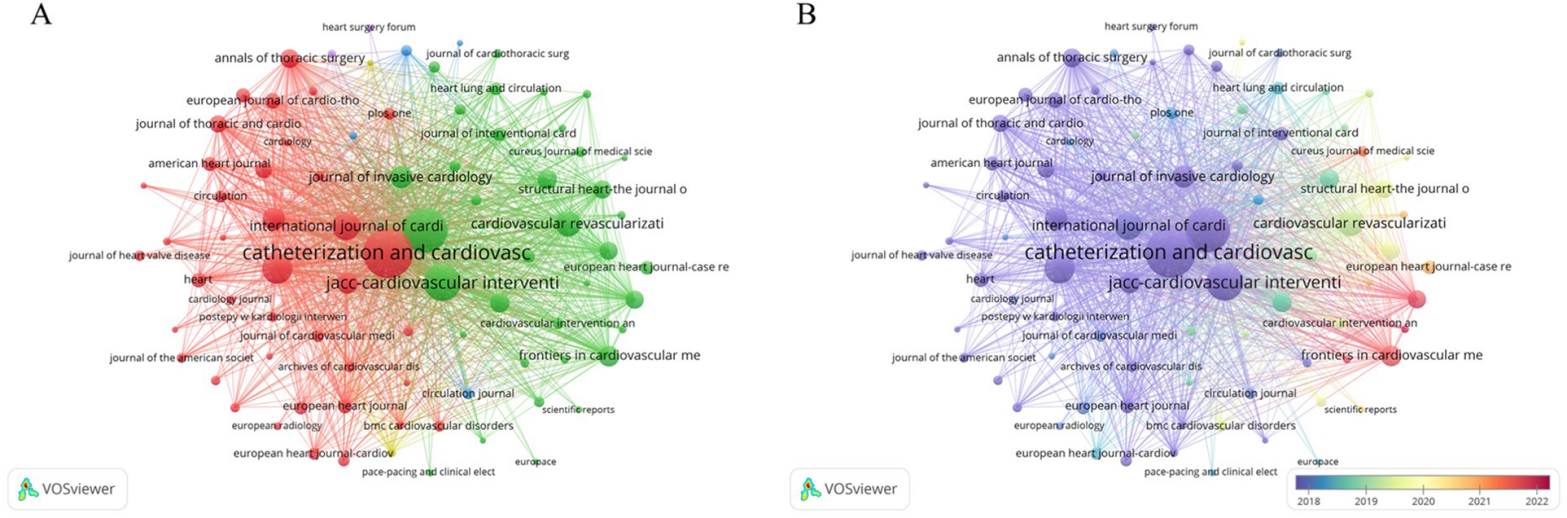
**(A)** Visual network map of 83 journals featured more than 20 publications within this area. Different colors indicate different themes of the publications, and the node size denotes the number of publications. Lines between nodes stand for relevance between various publications. **(B)** The timeline of visual network map with 83 journals featured more than 20 publications within this area. The warmer the color, the newer the publication, and the larger the node size, the more publications. Lines between nodes stand for relevance between various publications.

Furthermore, a total of 83 journals featured more than 20 publications within this field (Figure 5A). Notably, emerging journals such as “Frontiers in Cardiovascular Medicine” and “Cardiovascular Intervention and Therapeutics” have gained prominence by publishing papers in this evolving field (Figure 5B).

### Top cited publications

**Figure 6:**
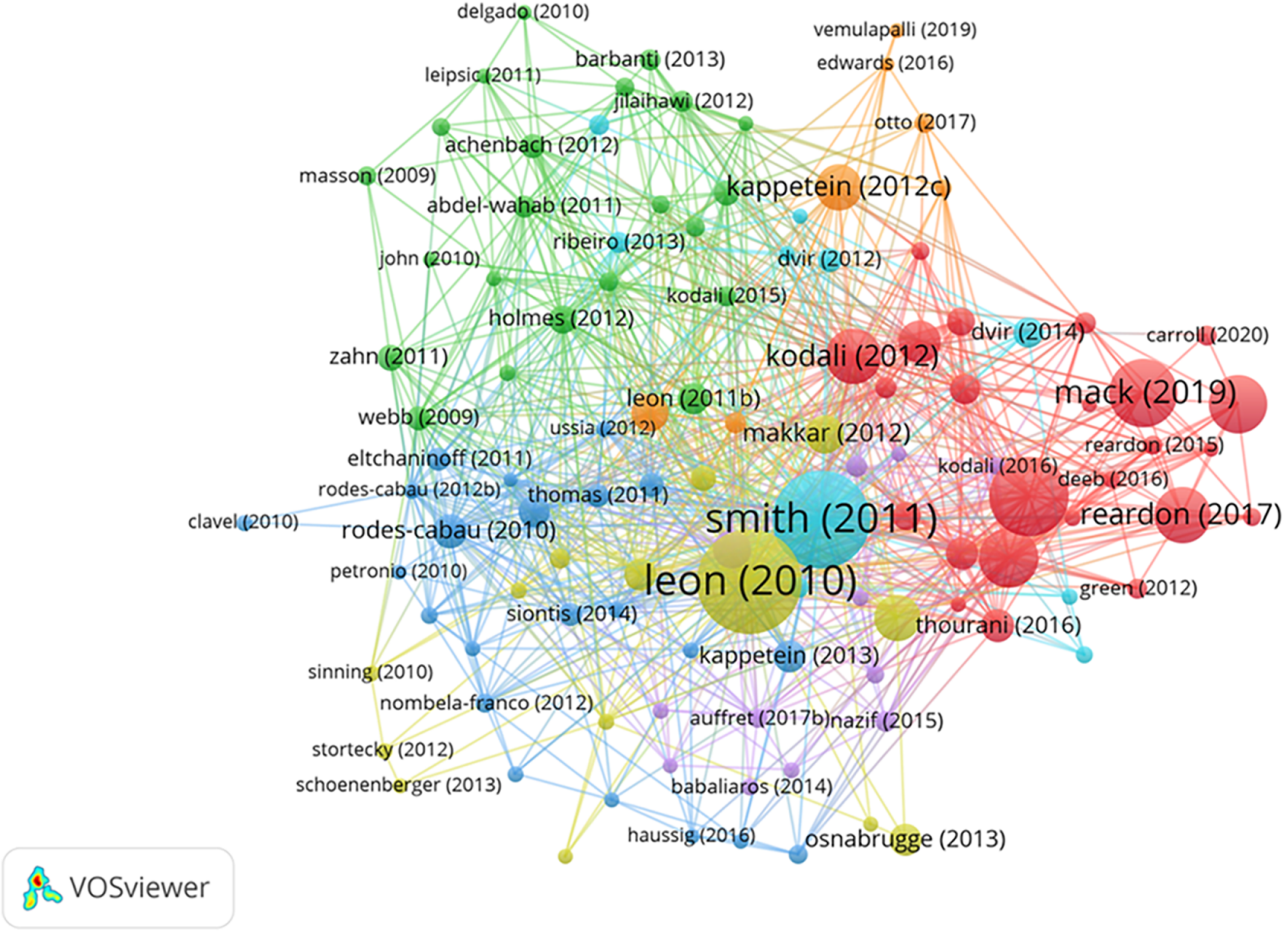
Visual analysis of 112 papers with more than 200 citations in this field. Different colors indicate different themes of the publications, and the node size denotes the number of citations. Lines between nodes stand for relevance between various publications.

Among the extensive array of publications, a notable 112 papers have accumulated more than 200 citations (Figure 6). The top twenty most cited papers are spearheaded by a paper titled “Transcatheter Aortic-Valve Implantation for Aortic Stenosis in Patients Who Cannot Undergo Surgery” amassing an impressive 5,400 citations. While, the paper titled “Transcatheter Aortic-Valve Replacement with a Balloon-Expandable Valve in Low-Risk Patients” stands out at the forefront, boasting an exceptional average citation count of 665.75 (Table 5).

**Table 5:** The top twenty cited publications related to TAVR.

Rank	Authors	Article title	Journal	Type	Times cited	Publication year	DOI link	Pubmed ID	Averaged time cited
1	Leon, MB et al.	Transcatheter Aortic-Valve Implantation for Aortic Stenosis in Patients Who Cannot Undergo Surgery.	New England Journal of Medicine	Article	5,400	2010	doi: 10.1056/NEJMoa1008232	20961243	415.38
2	Smith, CR et al.	Transcatheter versus Surgical Aortic-Valve Replacement in High-Risk Patients	New England Journal of Medicine	Article	4,802	2011	doi: 10.1056/NEJMoa1103510	21639811	400.17
3	Leon, MB et al.	Transcatheter or Surgical Aortic-Valve Replacement in Intermediate-Risk Patients	New England Journal of Medicine	Article	3,374	2016	doi: 10.1056/NEJMoa1514616	27040324	482.00
4	Mack, MJ et al.	Transcatheter Aortic-Valve Replacement with a Balloon-Expandable Valve in Low-Risk Patients	New England Journal of Medicine	Article	2,663	2019	doi: 10.1056/NEJMoa1814052	30883058	665.75
5	Adams, DH et al.	Transcatheter Aortic-Valve Replacement with a Self-Expanding Prosthesis	New England Journal of Medicine	Article	2,067	2014	doi: 10.1056/NEJMoa1400590	24678937	229.67
6	Popma, JJ et al.	Transcatheter Aortic-Valve Replacement with a Self-Expanding Valve in Low-Risk Patients	New England Journal of Medicine	Article	2,058	2019	doi: 10.1056/NEJMoa1816885	30883053	514.50
7	Reardon, MJ et al.	Surgical or Transcatheter Aortic-Valve Replacement in Intermediate-Risk Patients	New England Journal of Medicine	Article	1,895	2017	doi: 10.1056/NEJMoa1700456	28304219	315.83
8	Kodali, SK et al.	Two-Year Outcomes after Transcatheter or Surgical Aortic-Valve Replacement	New England Journal of Medicine	Article	1,834	2012	doi: 10.1056/NEJMoa1200384	22443479	166.73
9	Kappetein, AP et al.	Updated Standardized Endpoint Definitions for Transcatheter Aortic Valve Implantation the Valve Academic Research Consortium-2 Consensus Document	Journal Of the American College of Cardiology	Review	1,363	2012	doi: 10.1016/j.jacc.2012.09.001	23036636	123.91
10	Kappetein, AP et al.	Updated standardized endpoint definitions for transcatheter aortic valve implantation: the Valve Academic Research Consortium-2 consensus document	European Heart Journal	Article	1,380	2012	doi: 10.1093/eurheartj/ehs255	23026477	125.45
11	Mack, MJ et al.	5-year outcomes of transcatheter aortic valve replacement or surgical aortic valve replacement for high surgical risk patients with aortic stenosis (PARTNER 1): a randomised controlled trial	Lancet	Article	1,196	2015	doi: 10.1016/S0140-6736(15)60308-7	25788234	149.50
12	Makkar, RR et al.	Transcatheter Aortic-Valve Replacement for Inoperable Severe Aortic Stenosis	New England Journal of Medicine	Article	1,021	2012	doi: 10.1056/NEJMoa1202277	22443478	92.82
13	Gilard, M et al.	Registry of Transcatheter Aortic-Valve Implantation in High-Risk Patients	New England Journal of Medicine	Article	1,001	2012	doi: 10.1056/NEJMoa1114705	22551129	91.00
14	Tamburino, C et al.	Incidence and Predictors of Early and Late Mortality After Transcatheter Aortic Valve Implantation in 663 Patients with Severe Aortic Stenosis	Circulation	Article	958	2011	doi: 10.1161/CIRCULATIONAHA.110.946533	21220731	79.83
15	Rodes-Cabau, J et al.	Transcatheter Aortic Valve Implantation for the Treatment of Severe Symptomatic Aortic Stenosis in Patients at Very High or Prohibitive Surgical Risk Acute and Late Outcomes of the Multicenter Canadian Experience	Journal Of the American College of Cardiology	Article	836	2010	doi: 10.1016/j.jacc.2009.12.014	20096533	64.31
16	Thourani, VH et al.	Transcatheter aortic valve replacement versus surgical valve replacement in intermediate-risk patients: a propensity score analysis	Lancet	Article	789	2016	doi: 10.1016/S0140-6736 (16)30073-3	27053442	112.71
17	Popma, JJ et al.	Transcatheter Aortic Valve Replacement Using a Self-Expanding Bioprosthesis in Patients with Severe Aortic Stenosis at Extreme Risk for Surgery	Journal Of the American College of Cardiology	Article	778	2014	doi: 10.1016/j.jacc.2014.02.556	24657695	86.44
18	Osnabrugge, RLJ et al.	Aortic Stenosis in the Elderly Disease Prevalence and Number of Candidates for Transcatheter Aortic Valve Replacement: A Meta-Analysis and Modeling Study	Journal Of the American College of Cardiology	Article	765	2013	doi: 10.1016/j.jacc.2013.05.015	23727214	76.50
19	Moat, NE et al.	Long-Term Outcomes After Transcatheter Aortic Valve Implantation in High-Risk Patients with Severe Aortic Stenosis the UK TAVI (United Kingdom Transcatheter Aortic Valve Implantation) Registry	Journal of the American College of Cardiology	Article	750	2011	doi: 10.1016/j.jacc.2011.08.050	22019110	62.50
20	Kappetein, AP et al.	Updated standardized endpoint definitions for transcatheter aortic valve implantation: The Valve Academic Research Consortium-2 consensus document	Journal of Thoracic and Cardiovascular Surgery	Article	743	2013	doi: 10.1016/j.jtcvs.2012.09.002	23084102	74.30

Interestingly, these top cited twenty papers comprise a mixture of 19 articles and 1 review. Notably, 10 of these influential papers published in the *New England Journal of Medicine*, 5 were published in the Journal of the *American College of Cardiology*, the *Lancet* contributed 2 papers, and the *European Heart Journal*, *Circulation*, and the *Journal of Thoracic and Cardiovascular Surgery* each contributed one paper. Furthermore, it's worth noting that two of these highly impactful papers were published as recently as 2019, underscoring the continued relevance and significance of recent contributions in this field (Table 5).

### Evolution of keywords

A comprehensive set of 8,660 keywords were extracted from all 8,359 publications for co-occurrence analysis using Vosviewer. “stenosis”, “replacement”, “implantation,” “outcomes,” and “transcatheter” were ranked from first to fifth, featuring frequencies of 3,338, 2,893, 2,428, 2,258, and 2,048, respectively among the top 20 high-frequency keywords (Table 6).

**Table 6:** Top 20 highest frequency keywords in the publications related to TAVR.

Order	Keyword	Occurrences	Total link strength
1	Stenosis	3,338	28,423
2	Replacement	2,893	23,106
3	Implantation	2,428	19,447
4	Outcomes	2,258	18,770
5	Transcatheter Aortic Valve Replacement	2,048	16,577
6	Aortic Stenosis	1,922	16,357
7	Transcatheter Aortic Valve Implantation	1,706	13,988
8	Tavi	1,367	11,181
9	Impact	1,311	11,700
10	Risk	1,165	9,869
11	Mortality	1,151	10,198
12	Tavr	1,073	8,629
13	Predictors	1,016	9,438
14	Management	824	7,108
15	High-Risk Patients	766	7,292
16	Clinical-Outcomes	702	6,905
17	Surgery	637	5,175
18	Aortic valve stenosis	635	5,213
19	Societyfb	561	4,904
20	Regurgitation	543	5,001

Np, number of publications; Nc, number of citations; ACN, average cited number; IF, impact factor; JCR, journal cited report.

**Figure 7:**
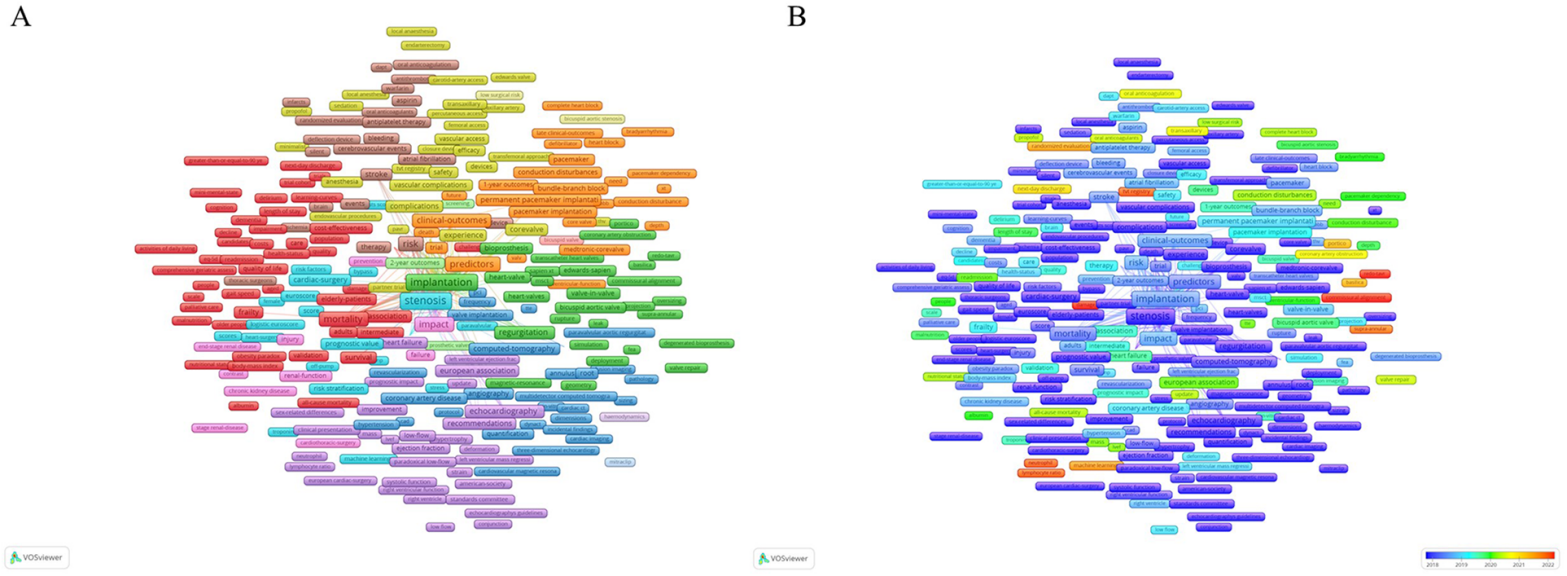
**(A,B)** Network map of high-frequency keywords that occur more than 10 times. A larger node size denotes a higher frequency of keyword occurrence. Different colors represent different clusters, and the lines between different nodes stand for the relationship between different keywords.

To gain deeper insights and understanding, all keywords with more than 10 occurrences were classified into 10 distinct clusters which enables a structured exploration of the interrelated themes within this vast body of literature using Vosviewer (Figure 7A).

To discern the evolving trends and themes of these keywords over time, a timeline visualization of these keywords was presented in Figure 7B. Notably, keywords such as “balloon-expandable valve” [average year published (AYP): 2021.55, Occurrence: 11], “coronary access” (AYP: 2021.47, Occurrence: 32), “next-day discharge” (AYP: 2020.39, Occurrence: 26), and “conduction disturbances” (AYP: 2021.31, Occurrence: 140) have emerged as notable keywords, suggesting their increasing importance and relevance within the field of study. This dynamic visualization aids in tracking the evolving landscape of research interests and priorities over time.

**Figure 8:**
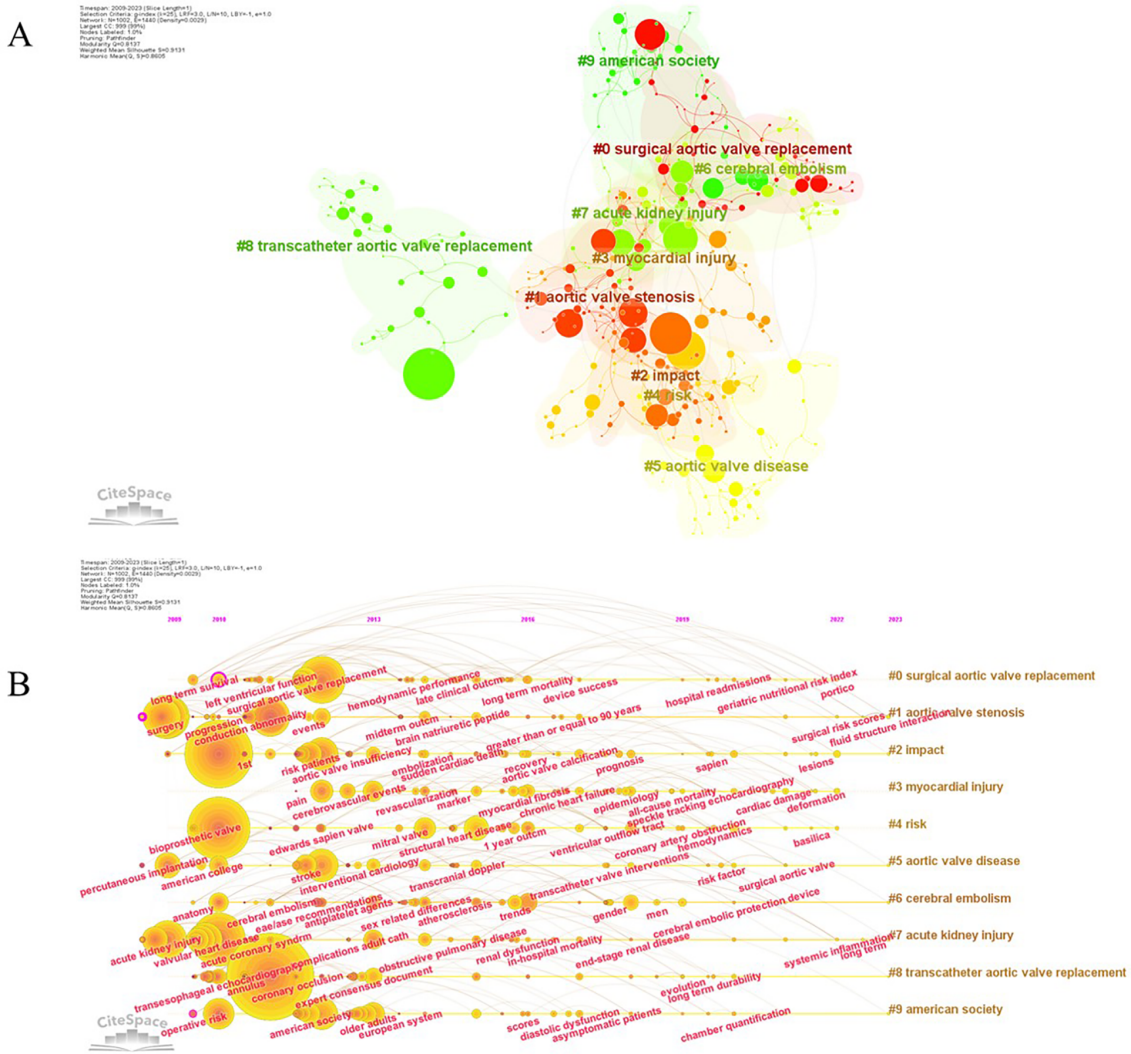
**(A)** All keywords were clarified into ten different clusters named by the keyword with the highest frequency. **(B)** The evolution of keywords analyzed by CiteSpace.

Based on Citespace, all keywords have been categorized into 10 distinct clusters, each named by the highest frequency in that cluster (Figure 8A). These clusters provide a structured representation of the research themes within the field: Cluster #0: Surgical Aortic Valve Replacement, Cluster #1: Aortic Valve Stenosis, Cluster #2: Impact, Cluster #3: Myocardial Injury, Cluster #4: Risk, Cluster #5: Aortic Valve Disease, Cluster #6: Cerebral Embolism, Cluster #7: Acute Kidney Injury, Cluster #8: Transcatheter Aortic Valve Replacement, Cluster #9: American Society. The timeline visualization of keywords within each cluster provides insights into the evolution of research themes over time. Early studies focused on topics such as long-term survival, conduction abnormalities, bioprosthetic valves, and transesophageal echocardiography. However, in the recent three years, researchers have shown increasing interest in keywords such as the geriatric nutritional risk index, surgical risk scores, cardiac damage, systemic inflammation, and chamber quantification (Figure 8B). These evolving themes reflect the dynamic nature of research within the field and highlight emerging areas of interest and investigation.

### Identification of research frontiers

To gain further insights into the research frontiers within this field, the top 100 keywords with the highest burst intensity and their corresponding burst years were identified by CiteSpace (Table 7), which reveals the evolution of research focus and provides valuable clues about emerging trends.

**Table 7:** Top 100 keywords with the with the strongest citation bursts.

Top 100 keywords with the strongest citation bursts					
Keywords	Year	Strength	Begin	End	2009–2023
High risk patients	2009	120.54	2009	2015	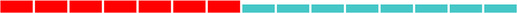
Prosthesis	2009	87.32	2009	2014	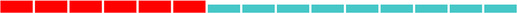
Replacement	2009	29.42	2009	2011	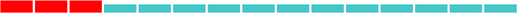
Experience	2009	21.17	2009	2013	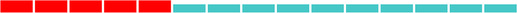
European society	2009	20.37	2009	2013	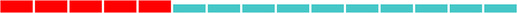
Surgery	2009	16.53	2009	2012	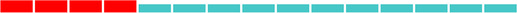
Heart valve	2009	16.5	2009	2012	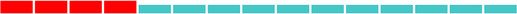
Feasibility	2009	16.41	2009	2012	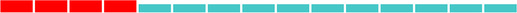
Elderly patients	2009	15.39	2009	2015	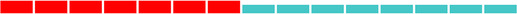
Retrograde	2009	13.63	2009	2014	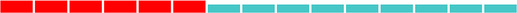
Stenosis	2009	11.14	2009	2012	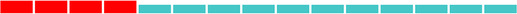
Octogenarians	2009	11.1	2009	2013	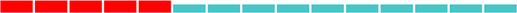
Percutaneous implantation	2009	10.89	2009	2013	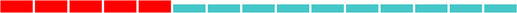
Percutaneous heart valve	2009	9.35	2009	2012	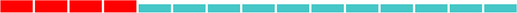
Natural history	2009	8.95	2009	2016	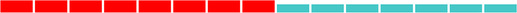
Acute renal failure	2009	8.7	2009	2016	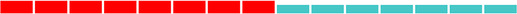
Guidelines	2009	8.07	2009	2012	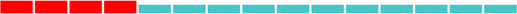
Euroscore	2009	7.53	2009	2013	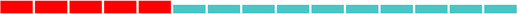
Valvuloplasty	2009	7.13	2009	2016	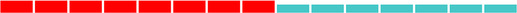
Position statement	2009	7.09	2009	2013	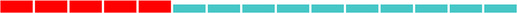
Valvular heart disease	2010	22.7	2010	2013	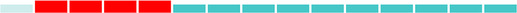
Follow up	2010	21.83	2010	2015	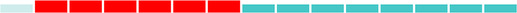
Root	2010	20.47	2010	2014	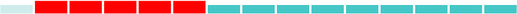
Multislice computed tomography	2010	19.58	2010	2015	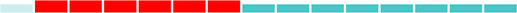
Echocardiography	2010	14.99	2010	2014	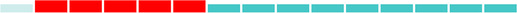
Transesophageal echocardiography	2010	13.26	2010	2014	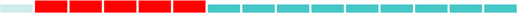
Corevalve revalving system	2010	12.36	2010	2016	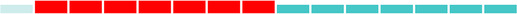
Aortic annulus	2010	9.86	2010	2014	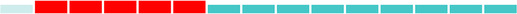
Success	2010	8.9	2010	2013	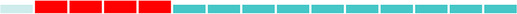
Bioprosthesis	2010	8.21	2010	2013	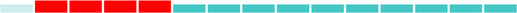
Doppler echocardiography	2010	6.89	2010	2016	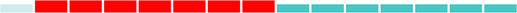
Requirement	2010	6.45	2010	2015	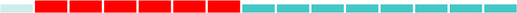
Corevalve	2010	21.14	2011	2014	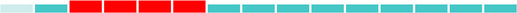
Transcatheter aortic valve implantation	2009	13.13	2011	2012	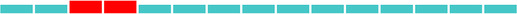
Permanent pacemaker requirement	2011	10.81	2011	2014	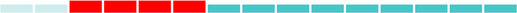
Edwards sapien (Tm)	2011	8.19	2011	2014	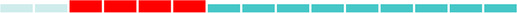
Late outcome	2011	8.04	2011	2015	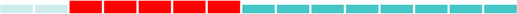
Dimensions	2011	7.31	2011	2015	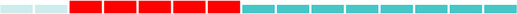
Quality of life	2011	6.8	2011	2013	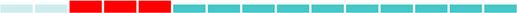
Device	2011	6.58	2011	2014	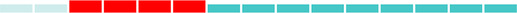
Heart valve prosthesis implantation	2011	6.39	2011	2013	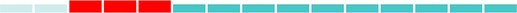
Outcome source registry	2012	25.52	2012	2015	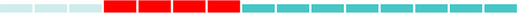
European registry	2012	24.91	2012	2015	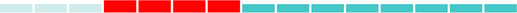
Academic research consortium	2011	18.17	2012	2015	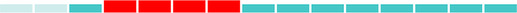
Interventions	2012	15.62	2012	2015	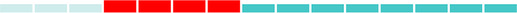
Predictive factors	2012	13.56	2012	2016	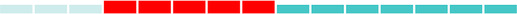
Multidetector computed tomography	2012	11.51	2012	2015	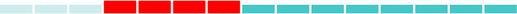
Edwards sapien valve	2012	10.32	2012	2015	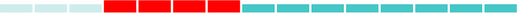
Eae/ase recommendations	2012	10.31	2012	2016	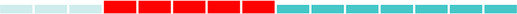
Annulus	2011	9.12	2012	2016	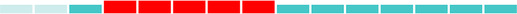
Valve implantation	2012	8.79	2012	2015	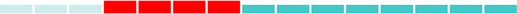
Implantation impact	2012	7.35	2012	2015	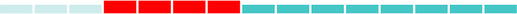
Clinical trials	2012	7.33	2012	2014	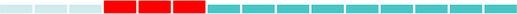
Paravalvular aortic regurgitation	2012	6.58	2012	2016	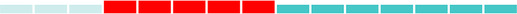
Consensus report	2012	6.37	2012	2015	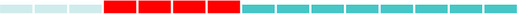
Clinical application	2012	6.37	2012	2015	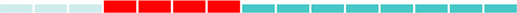
Surgical risk	2012	6.37	2012	2015	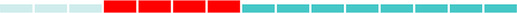
Edwards sapien	2011	20.63	2013	2016	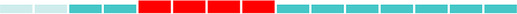
End point definitions	2012	11.7	2013	2014	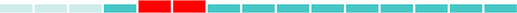
3-dimensional transesophageal echocardiography	2013	8.74	2013	2017	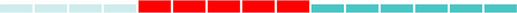
Cardiovascular magnetic resonance	2011	6.93	2013	2016	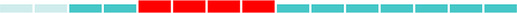
Standards committee	2013	6.54	2013	2014	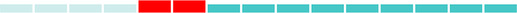
Treatment outcome	2013	6.28	2013	2016	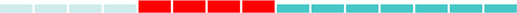
Consensus document	2013	6.32	2014	2017	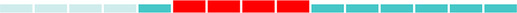
Long term outcome	2012	6.28	2014	2016	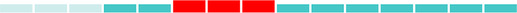
Registry	2011	23.66	2015	2018	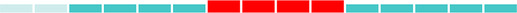
Multicenter	2012	9.55	2015	2017	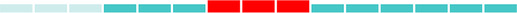
Edwards sapien Xt	2015	7.88	2015	2018	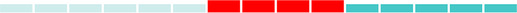
Placement	2013	7.64	2015	2017	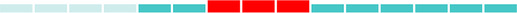
United States	2015	7.09	2015	2018	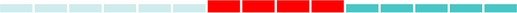
Repair	2015	6.53	2015	2019	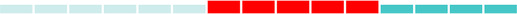
2 year outcome	2016	10.21	2016	2017	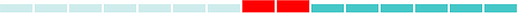
Partner trial	2014	8.41	2016	2018	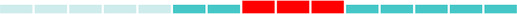
Local anesthesia	2014	6.5	2016	2019	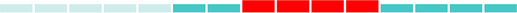
Intermediate risk patients	2016	38.42	2017	2018	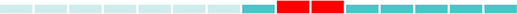
Risk patients	2012	18.48	2017	2018	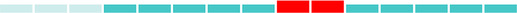
Trial	2013	12.33	2017	2018	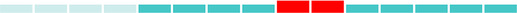
Meta-analysis	2012	10.55	2017	2018	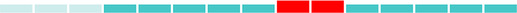
Intermediate risk	2013	8.73	2017	2020	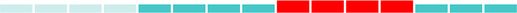
Early discharge	2017	7.2	2017	2019	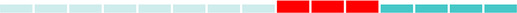
Subclinical leaflet thrombosis	2017	9.94	2018	2023	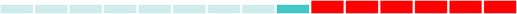
Protection	2018	7.16	2018	2021	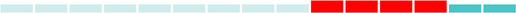
Leaflet thrombosis	2018	6.7	2018	2021	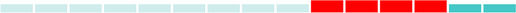
Thoracic surgeons/American college	2018	15.39	2019	2021	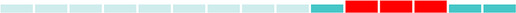
Coronary obstruction	2013	12.19	2019	2023	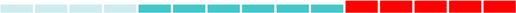
Classification	2017	7.31	2019	2023	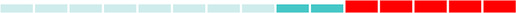
Conscious sedation	2018	6.58	2019	2021	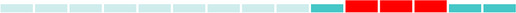
Balloon-expandable valve	2019	6.36	2019	2023	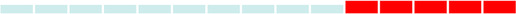
Cardiology esc	2010	6.22	2019	2023	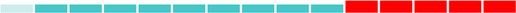
Case report	2018	16.14	2020	2023	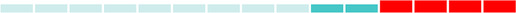
Association	2009	12.05	2020	2023	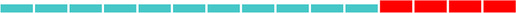
American society	2012	10.72	2020	2023	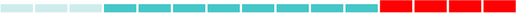
Bicuspid aortic valve	2009	9.46	2020	2021	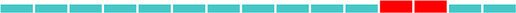
Coronary access	2020	9.13	2020	2023	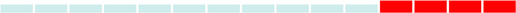
Update	2015	7.62	2020	2023	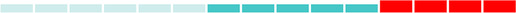
Tavr	2015	34.38	2021	2023	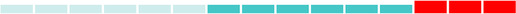
Conduction disturbances	2013	18.85	2021	2023	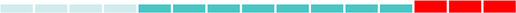
Insights	2014	7.92	2021	2023	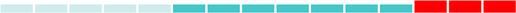
Next day discharge	2021	7.34	2021	2023	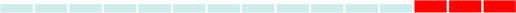
American college	2010	6.2	2021	2023	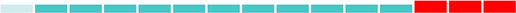

In the early stages of TAVR research, the spotlight was on keywords such as “high-risk patients,” “prosthesis,” and “elderly patients.” “High-risk patients” exhibited the strongest burst with a burst strength of 120.54. This keyword signifies the foundational criterion for TAVR candidacy, emphasizing that initially, only high-risk patients who were not suitable for surgical interventions considered TAVR as a potential treatment option. It served as a defining standard for cardiologists during this period. Emerging keywords in recent years include: “Balloon-expandable valve” (Strength: 6.36, Burst Years: 2019–2023), “Coronary access” (Strength: 9.13, Burst Years: 2020–2023), “Next-day discharge” (Strength: 7.34, Burst Years: 2021–2023), “Conduction disturbances” (Strength: 18.85, Burst Years: 2021–2023), “Case report” (Strength: 16.14, Burst Years: 2020–2023), “Conscious sedation” (Strength: 6.58, Burst Years: 2019–2021), “Coronary obstruction” (Strength: 12.19, Burst Years: 2019–2023). These emerging keywords underscore the current research frontiers in the field of TAVR, highlighting areas of heightened interest and investigation. Notably, the focus has shifted towards procedural refinement, patient outcomes, and innovative techniques such as conscious sedation, as well as addressing specific challenges like coronary obstruction and conduction disturbances. This dynamic landscape reflects the evolving nature of TAVR research and its continued expansion into novel areas of inquiry.

## Discussion

To our knowledge, this work is the first bibliometric analysis to systematically review TAVR studies over the past 15 years. The salient findings are summarized as follows: 1. TAVR research is a dynamic and expanding area, as evidenced by escalating publication and citation numbers; 2. The United States exerts dominant influence in this sphere, evidenced by unparalleled counts of publications and citations; 3. “Catheterization and Cardiovascular Interventions” emerges as the preeminent journal for TAVR-related literature; 4. Emerging research foci within this field encompass topics such as balloon-expandable valves, strategies for coronary access, protocols for next-day discharge, and the management of conduction disturbances.

Our analysis reaffirms the sustained interest and growth in TAVR research, a fact underscored by the consistent increase in the quantity of publications and citations. In recent years, several seminal papers have played a pivotal role in shaping the field and contributing to this upward trajectory. The “Placement of Aortic Transcatheter Valves” (PARTNER) trial, led by Leon et al. is a landmark study that revolutionized the perception of TAVR (24). This pivotal research, cited extensively in subsequent studies, established TAVR as a valid alternative to SAVR for inoperable and high-risk patients. The PARTNER trial's long-term follow-up data, published by Adams et al. further solidified TAVR's position as a game-changer in aortic valve therapy (25). Another study completed by Leon et al. on TAVR in intermediate-risk patients expanded the horizons of TAVR applicability (26). This research demonstrated that TAVR could be performed with similar outcomes to SAVR in patients previously considered ineligible. This transformative finding paved the way for the inclusion of a broader patient population, further driving interest in TAVR research.

Rodes-Cabau et al. focused on complications in TAVR procedures has been widely cited due to its comprehensive analysis of adverse events and their management strategies. Understanding and mitigating complications are critical aspects of ongoing TAVR research, and this paper continues to guide efforts to enhance procedural safety (27). Yoon et al. explored the feasibility of TAVR in patients with bicuspid aortic valves has garnered significant attention, and addressed an evolving research hotspot and highlighted the expanding scope of TAVR applications, reflecting the field's ongoing growth (28). The cost-effectiveness and economic implications of TAVR vs. SAVR have been the subject of extensive research. Studies such as the analysis finished by Reynolds et al. have been instrumental in evaluating the economic feasibility of TAVR, influencing healthcare decision-makers and insurers (29). As healthcare systems worldwide grapple with resource allocation, such studies remain highly relevant and cited.

In the current study, we identified the United States as the leading country in TAVR research, with a substantial number of publications and citations. Recent research has provided insights into the factors contributing to this dominance. A study conducted by Garcia et al. attributes this leadership to the robust infrastructure of cardiovascular centers in the United States, enabling large-scale clinical trials and fostering innovation (30). Furthermore, the United States remains at the forefront of technological advancements in TAVR. Makkar and colleagues highlight ongoing efforts to develop next-generation balloon-expandable valves with improved hemodynamics and long-term durability. Collaborations between American researchers and medical device companies continue to drive innovation, reinforcing the nation's leadership in this arena (31). However, it is essential to underscore the global nature of TAVR research and the importance of international collaboration. The Multi-Ethnic TAVR (META-TAVR) Consortium, involving researchers from various countries, exemplifies the cooperative spirit in advancing TAVR science (32). Such collaborations facilitate knowledge exchange and contribute to the development of universal best practices.

The journal *Catheterization and Cardiovascular Interventions* was the primary publication platform for TAVR-related research. Recent studies continue to underscore the journal's central role in disseminating critical TAVR findings and articles published in this journal receive, on average, 20% more citations compared to those in other cardiovascular journals. Moreover, the journal remains committed to advancing the field through special issues and dedicated sections. The “TAVR Innovations” section, initiated in collaboration with leading TAVR experts, serves as a focal point for cutting-edge research. Researchers continue to leverage this platform to share novel techniques and outcomes, enriching the TAVR knowledge base. While “*Catheterization and Cardiovascular Interventions*” maintains its prominence, researchers should also consider submitting their work to other high-impact journals to ensure diverse dissemination and maximize their research's reach.

Although our analysis relied solely on the Web of Science Core Collection, several emerging research hotspots within the TAVR field were identified, each offering promising opportunities for advancing the procedure and improving patient outcomes.

(a) Balloon-Expandable Valve Technology

Balloon-expandable valve technology remains at the forefront of TAVR research and development. Recent work finished by Ielasi et al. introduces a novel valve design that incorporates advanced materials, enhancing its durability and biocompatibility (33). This innovation has the potential to reduce the need for repeat interventions and improve long-term valve function. Additionally, studies are exploring the role of artificial intelligence (AI) in optimizing valve sizing and positioning, which aims to enhance procedural precision and minimize paravalvular leakage. Such cutting-edge technologies represent the convergence of medicine and engineering in the pursuit of safer and more effective TAVR procedures (34).

(b) Coronary Access Management

Efficient coronary access management post-TAVR remains pivotal for ensuring myocardial perfusion and minimizing complications. Recently, Tang and colleagues investigated the utility of intravascular imaging techniques, such as optical coherence tomography (OCT), in assessing coronary ostia patency after valve deployment (35). This technology provides real-time, high-resolution images, which enabled precise evaluation and intervention. Moreover, advancements in robotic-assisted TAVR procedures, as highlighted by Baig et al., offer the potential to further enhance coronary access management, and the robotic systems provide unparalleled precision during valve implantation, reducing the risk of obstructing coronary arteries and simplifying complex procedures (36).

(c) Next-Day Discharge Protocols

The adoption of next-day discharge protocols continues to gain momentum as healthcare systems seek to optimize resource utilization without compromising patient care. Recent studies, such as the multicenter trial led by Butala et al. provide compelling evidence supporting the safety and feasibility of next-day discharge for carefully selected TAVR patients (37). These findings have significant implications for healthcare cost savings and resource allocation. Furthermore, investigations into remote monitoring and telemedicine for post-TAVR follow-up care have expanded. Tian and colleagues demonstrated the effectiveness of remote monitoring in detecting early complications, allowing timely intervention and reducing readmission rates (38). The integration of telemedicine into TAVR care pathways may prove instrumental in enhancing patient outcomes and streamlining healthcare delivery.

(d) Conduction Disturbances

Conduction disturbances during TAVR procedures remain a complex challenge, and recent research continues to explore strategies to mitigate their occurrence and improve patient safety. Vijayaraman et al. highlighted the potential benefits of His bundle pacing in preventing conduction disturbances (39). Furthermore, advancements in pre-procedural risk stratification have gained prominence. Schoechlin et al. identifies specific patient characteristics such as pre-existing bundle branch blocks, that may predispose individuals to conduction disturbances those results enabled more targeted monitoring and intervention strategies for at-risk patients (40).

## Conclusions

Investigation into TAVR has emerged as a major area of scholarly focus, evidenced by a pronounced rise in both publications and citations. The United States stands at the forefront of this research, leading internationally in the volume of TAVR-related publications and citations. Within this landscape, Columbia University ranks as the preeminent institution in terms of publication output, with the Cleveland Clinic Foundation and Harvard University also contributing significantly. Key research themes such as “balloon-expandable valve,” “coronary access,” “next-day discharge,” “conduction disturbances,” “case report,” and “coronary obstruction” are shaping up as current and prospective research hotspots, signaling potential areas for future study and innovation.

## Data Availability

The original contributions presented in the study are included in the article/Supplementary Material, further inquiries can be directed to the corresponding authors.

## Supplementary material

Supplementary Figure 1 The flow chart of current study.
