# Supplementary figures and images for: Current hotspot and study trend of transcatheter aortic valve replacement, a bibliometric analysis from 2009 to 2023
Source: Front Cardiovasc Med. 2025 Apr 14;12:1411561. doi: 10.3389/fcvm.2025.1411561 (PMC12034703; doi:10.3389/fcvm.2025.1411561)

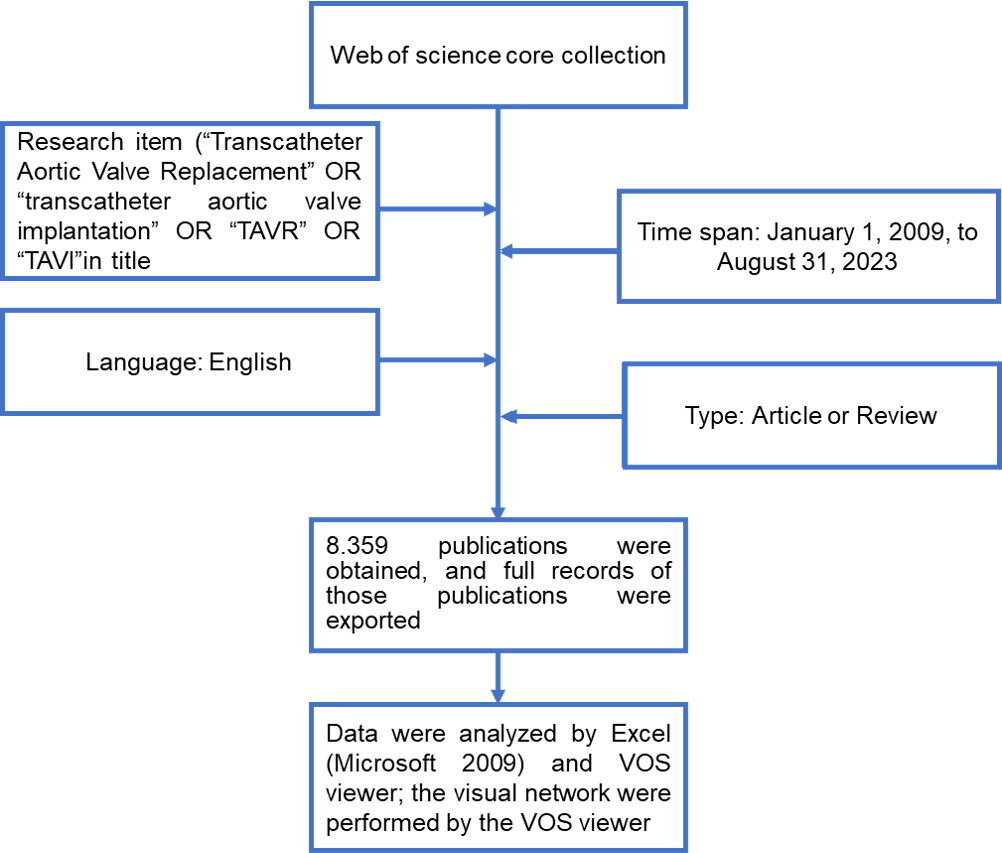

Supplement: Supplementary Figure 1 — The flow chart of current study. [file Image1.tif]
